# Abnormal regulation of BCR signalling by c-Cbl in chronic lymphocytic leukaemia

**DOI:** 10.18632/oncotarget.25951

**Published:** 2018-08-14

**Authors:** Veronica Martini, Federica Frezzato, Filippo Severin, Flavia Raggi, Valentina Trimarco, Leonardo Martinello, Rosa Molfetta, Andrea Visentin, Monica Facco, Gianpietro Semenzato, Rossella Paolini, Livio Trentin

**Affiliations:** ^1^ Department of Medicine, Hematology and Clinical Immunology Branch, University School of Medicine, Padua, Italy; ^2^ Venetian Institute of Molecular Medicine, VIMM, Padua, Italy; ^3^ Department of Molecular Medicine, University of La Sapienza, Rome, Italy

**Keywords:** CIN85, Lyn, PI3Kp85, cortactin

## Abstract

Abnormalities of molecules involved in signal transduction pathways are connected to Chronic Lymphocytic Leukemia (CLL) pathogenesis and a critical role has been already ascribed to B-Cell Receptor (BCR)-Lyn axis. E3 ubiquitin ligase c-Cbl, working together with adapter protein CIN85, controls the degradation of protein kinases involved in BCR signaling. To investigate cell homeostasis in CLL, we studied c-Cbl since in normal B cells it is involved in the ubiquitin-dependent Lyn degradation and in the down-regulation of BCR signaling. We found that c-Cbl is overexpressed and not ubiquitinated after BCR engagement. We observed that c-Cbl did not associate to CIN85 in CLL with respect to normal B cells at steady state, nor following BCR engagement. c-Cbl association to Lyn was not detectable in CLL after BCR stimulation, as it happens in normal B cells. In some CLL patients, c-Cbl is constitutively phosphorylated at Y731 and in the same subjects, it associated to regulatory subunit p85 of PI3K. Moreover, c-Cbl is constitutive associated to Cortactin in those CLL patients presenting Cortactin overexpression and bad prognosis. These results support the hypothesis that c-Cbl, rather than E3 ligase activity, could have an adaptor function in turn influencing cell homeostasis in CLL.

## INTRODUCTION

B cell receptor (BCR) signaling is now recognized as a central pathway in chronic lymphocytic leukemia (CLL) pathogenesis [1] basing on preclinical studies that demonstrated the importance of BCR activation for survival and proliferation of CLL cells *in vitro* [2], and in CLL mouse models [3]. BCR triggering activates a complex signaling cascade, including upstream kinases, such as the SRC-kinase LYN, SYK, BTK, and PI3Kδ, which transduce signals to cytoskeletal activators, such as hematopoietic lineage cell-specific Lyn-substrate 1 (HS1) [4] and Cortactin [5], and downstream effectors, including AKT and ERK kinases [6]. A large number of CLL patients treated with BTK or PI3Kδ inhibitors (i.e. Ibrutinib and idelalisib), which primarily target BCR signaling, achieve durable responses, corroborating the relevance of BCR signaling in CLL pathogenesis [7]. In CLL, little is known on the alterations affecting the mechanisms involved in the preservation of homeostasis of BCR signaling. Constitutive activation of Syk/Lyn, PI3K/Akt and PLCγ2/PKC in CLL B cells raises the question of their failure. Much interest has recently been focused on c-Cbl (c-Casitas B lineage lymphoma) a proto-oncogene that modulates receptor tyrosine kinase (RTK) as well as cytoplasmic tyrosine kinase activity thanks to its ability to bind, through its tyrosine kinase binding domain (TKB) and its proline-rich domain, multiple Src homology 3 (SH3)-containing partners, such as Lyn, Btk and the BLNK [8]. In particular, following recruitment to phosphorylated tyrosine kinase, c-Cbl is also phosphorylated at multiple tyrosine residues, and provides docking sites for the SH2 domains of Vav (Y700), Crkl (Y700 and Y774) and the p85 regulatory subunit of PI3K (Y731), promoting cell survival and proliferation through these interactions [9]. Data from the literature report that c-Cbl is overexpressed and significantly hypophosphorylated at Y700 in progressive disease CLL patients [10]. c-Cbl phosphorylation at Y371, located in a linker region between the RING finger and the TKB domain, releases c-Cbl from its autoinhibited structure by triggering a conformational change that leads to an enhanced transfer of ubiquitin from the E2 enzyme to the substrate proteins. Sporadic and germline c-CBL mutations have been already identified in JMML (Juvenile Myelomonocytic Leukemia) patients with the emerging of Y371H mutation which results in the loss of Cbl’s ubiquitin ligase function [11]. Moreover, cells expressing c-CblY371H are chemoresistant due to hyperactive Src kinase that promotes AKT signaling via enhanced binding of c-Cbl(Y371H) to the PI3K regulatory subunit p85. Various evidence has highlighted that c-Cbl is involved both in positive and negative regulation of signals originated from a number of receptors, including immunoreceptors and receptors for growth factors, death signals, hormones, cytokines, cell–cell contacts, or cell–substrate contacts [12]. The implications are that c-Cbl participates in numerous processes, such as survival or differentiation, and operates at different cell developmental stages. Based on these observations, herein, we investigated the expression and the role of c-Cbl in CLL B cells. Based on our finding it seems unlikely that Cbl plays a role as E3 ligase in CLL, but rather as adaptor protein supporting pro-survival signaling from BCR.

## RESULTS

### c-Cbl is overexpressed and is not associated to the adaptor protein CIN85 in neoplastic B lymphocytes

**Figure 1:**
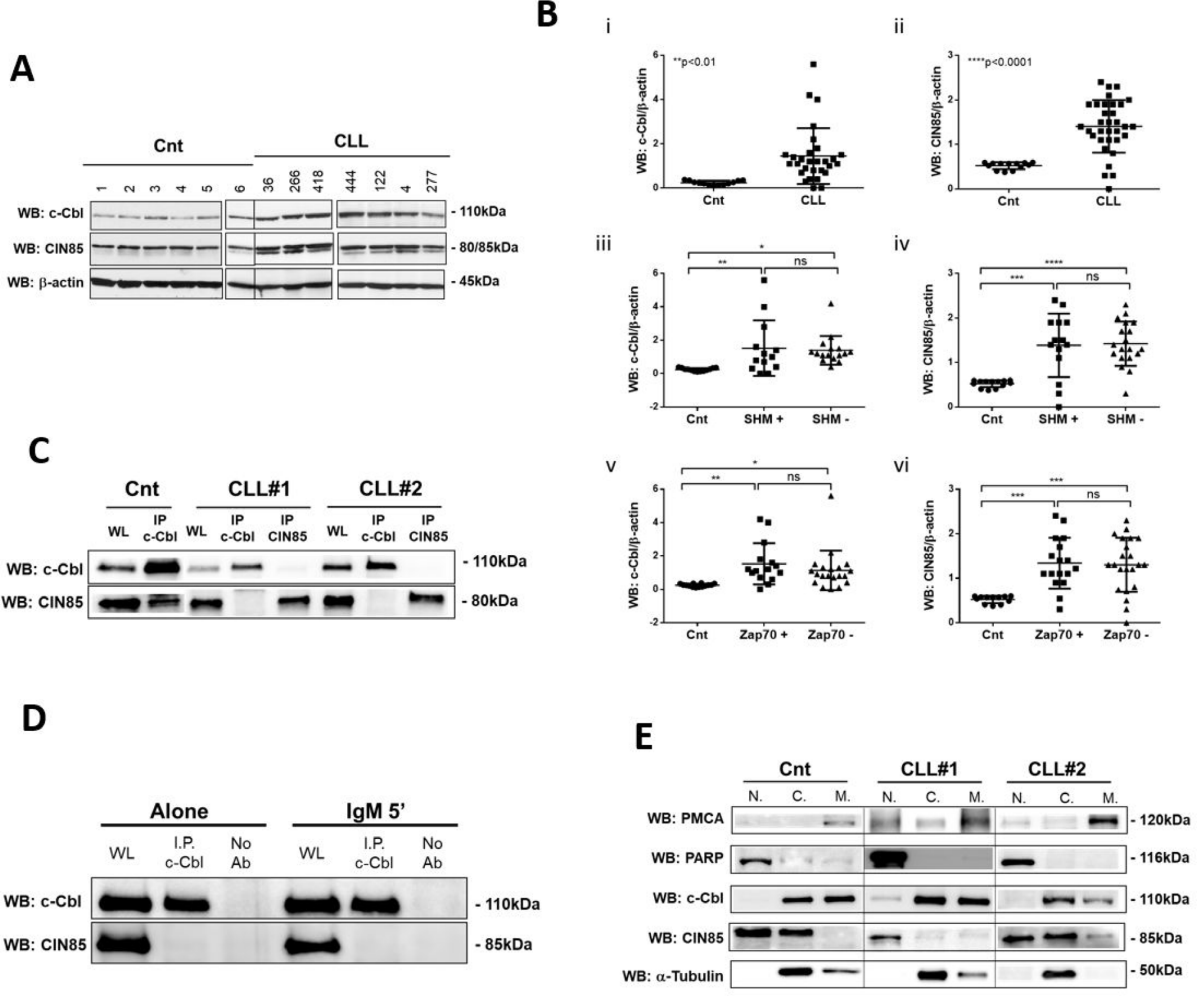
Evaluation of c-Cbl and CIN85 expression (**A**) Western blotting (7.5%Acrylamide/Bis-acrylamide) of 7 CLL and 6 normal B lymphocytes is representative of all samples analysed (CLL = 40 and Cnt = 13). The lysates obtained from normal B lymphocytes and leukemic B cells from CLL patients were analyzed by immunostaining with antibody against c-Cbl and CIN85. Blots were reprobed with anti-β-actin antibody as loading control. (**B**) Figure represents the densitometric value (arbitrary unit) of (i) c-Cbl/β-actin and (ii) CIN85/β-actin ratio of B cells samples of 13 healthy subjects (Cnt) and 40 CLL patients (CLL). Data obtained were evaluated for their statistical significance with the Student’s *t* test (^**^p < 0.01 and ^****^p < 0.0001 between normal controls and CLL patients). Data obtained were correlated with CLL prognostic factors: normal *vs* mutated *vs* unmutated for (iii) c-Cbl (^**^p < 0.01; ^*^p < 0.05; ns; Student’s *t* test) and for (iv) CIN85 (^***^p < 0.001; ^****^p < 0.0001; ns; Student’s *t* test); normal *vs* ZAP70+ *vs* ZAP70– for c-Cbl (^**^p < 0.01; ^*^p < 0.05; ns; Student’s *t* test) and for CIN85 (^***^p < 0.001; ^***^p < 0.001; ns; Student’s *t* test). (**C**) The lysates obtained from normal B lymphocytes (Cnt) and leukemic B cells from CLL patients (CLL#1 and CLL#2) were immunoprecipitated with anti-c-Cbl or anti-CIN85 antibody. Immunocomplexes were loaded in SDS-PAGE (10% Acrylamide/Bis-acrylamide) and then probed with anti-c-Cbl and anti–CIN85 antibody. (**D**) The lysates obtained from 10 leukemic B cells before and after 5 minutes with IgM stimuli (10 ng/mL) were immunoprecipitated with anti-c-Cbl. Immunocomplexes were loaded in SDS-PAGE (10% Acrylamide/Bis-acrylamide) and then probed with anti-c-Cbl and anti–CIN85. **(E**) Aliquots of B cells from healthy subjects (Cnt) or CLL patients have been processed with a cell-fractionation kit according to the manufacturer’s instruction and then comparable aliquots of the different fractions (nuclei = N, cytosols = C and membrane = M) were loaded on SDS-PAGE and the separated proteins were immunostained with anti-c-Cbl, anti-CIN85, anti-tubulin (cytosolic marker), anti-PMCA (membrane marker) and anti-PARP (nuclear marker) antibodies. Figure is representative of different experiments performed on total 13 CLL patients and 4 normal controls. WL: Whole Lysate; I.P.: ImmunoPrecipitation; Ab: Antibody.

In normal B cells, c-Cbl and CIN85 are poorly expressed and constitutively associated and this association is low in unstimulated cells, but increased after BCR activation [13]. CIN85 is an adapter molecule required for c-Cbl-mediated regulation of BCR signaling [14]. Expression levels of both c-Cbl and CIN85 were measured by WB analysis in purified B cells for 40 therapy free CLL patients and 13 healthy controls (Figure 1A). We found that both c-Cbl and CIN85 were significantly increased in CLL patients with respect to normal controls (c-Cbl ^**^p < 0.01, CIN85 ^****^p < 0.0001; Figure 1B i-ii); no differences were observed between the two prognostic groups of CLL patients (SHM+/− and Zap70+/−; Figure 1B iii-vi). By co-immunoprecipitation experiments, we found that, differently from what has been described for normal B cells [13, 14] (Supplementary Figure 1), CIN85 and c-Cbl were not constitutive associated in neoplastic B lymphocytes (compare Cnt *n* = 3 with CLL *n* = 20; CLL#1 and CLL#2; Figure 1C), neither upon anti-IgM BCR stimulation (Figure 1D). This lack of association is independent from the subcellular redistribution of c-Cbl and CIN85. In fact, even when the two proteins were both found in the cytosol (*n* = 8; i.e. CLL#2; Figure 1E), they did not co-immunoprecipitate. We highlighted that in *n* = 8 CLL patients CIN85 is expressed only in the nucleus (i.e. CLL#1; Figure 1E) and in *n* = 2 CLL patients is expressed only in the cytosol (data not show). This is a hallmark of neoplastic lymphocytes, since in normal B lymphocytes CIN85 is distributed both in the nucleus and cytosol (*n* = 3; i.e. Cnt; Figure 1E).

A possible explanation about the lack of interaction between CIN85 and c-Cbl, is that CIN85 in neoplastic B cells presents two bands with a molecular weight of about 80/85kDa, instead in normal controls it is evident only one (CIN85 lane; Figure 1A). This is a peculiarity of CLL patients and could influence the binding of CIN85 to its partners, i.e. c-Cbl.

All together these finding demonstrate that in neoplastic B lymphocytes Cbl and CIN85 are overexpressed but not present as a constitutive or inducible molecular complex.

### Lyn is not ubiquitinated after BCR engagement in neoplastic B cells

**Figure 2:**
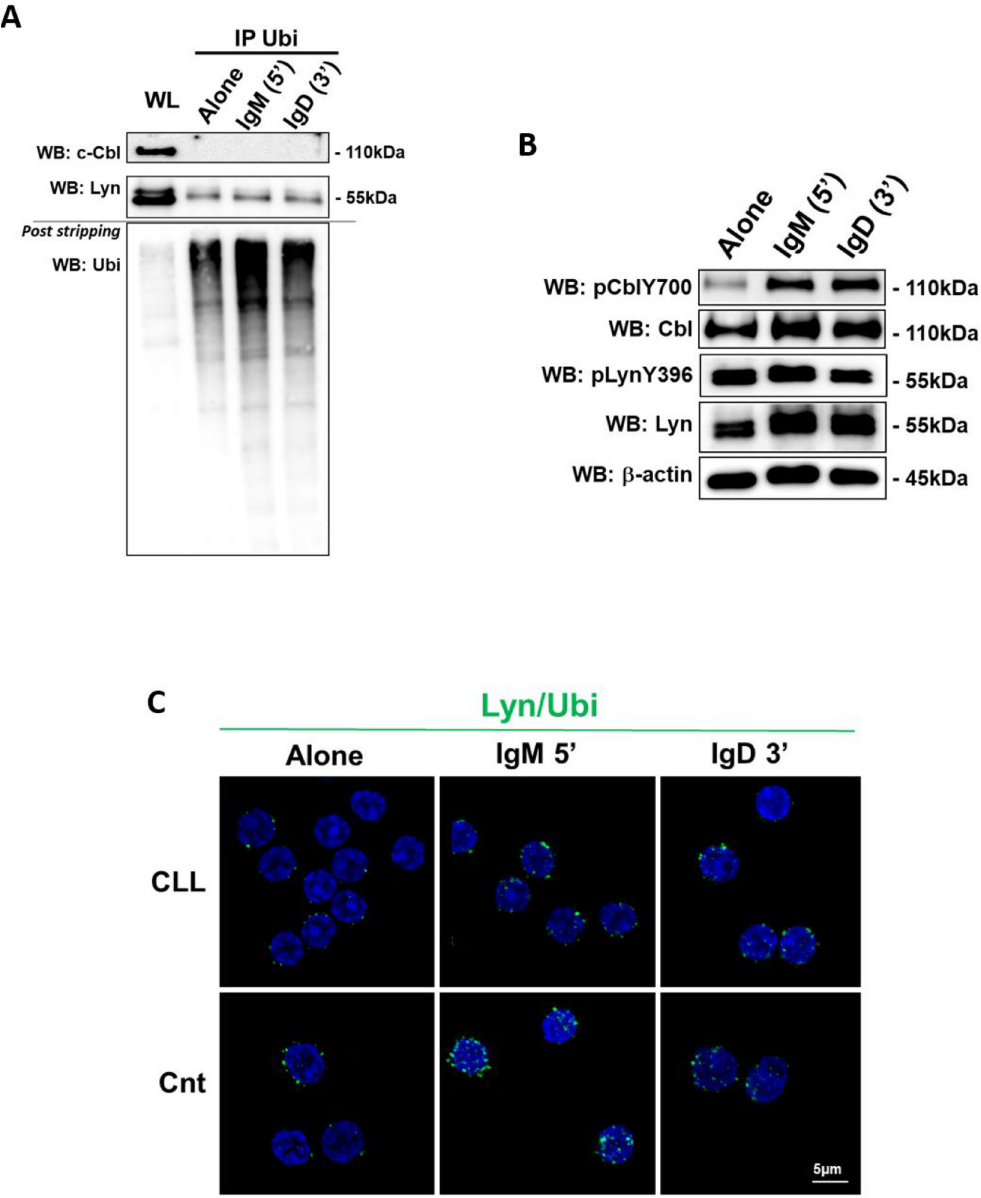
Lyn and c-Cbl ubiquitination (**A**) The lysates obtained from leukemic B cells of CLL patients (*n* = 10), before and after 5 minutes of IgM and 3 minutes of IgD stimuli (10ng/mL), were immunoprecipitated with anti-Ubiquitin antibody (IP Ubi). Immunocomplexes (IP) and whole lysate (WL) were loaded in SDS-PAGE (10% Acrylamide/Bis-acrylamide) and then probed with anti-c-Cbl, anti–Lyn and, after stripping the membrane, with anti-ubiquitin-HRP supplied in ubiquitin-immunoprecipitation kit. (**B**) Western blotting (10% Acrylamide/Bis-acrylamide) of CLL B lymphocytes is representative of all 10 samples analysed. The lysates obtained from leukemic B cells, before and after IgM and IgD stimuli, were analyzed by immunostaining with antibody against pCblY700, c-Cbl, pLynY396, Lyn and β-actin. (**C**) Proximity ligation assay (PLA) was performed on CLL and normal B cells (Cnt). Rabbit polyclonal anti-Lyn and mouse monoclonal anti-ubiquitin were used as primary Abs. Positive PLA signals are visualized as green fluorescent spots, nuclei are in blue (DAPI). Images are representative of three independent experiments, and were acquired with zoom 3 using 60X/1.35NA oil immersion objective. Z-projections of 15 slices are shown. Scale bar: 5 µm.

In normal B cells, protein homeostasis is regulated by ubiquitination events and, in particular, after BCR activation, Lyn and c-Cbl undergo ubiquitination and proteasomal degradation [15–17]. It has been demonstrated both *in vivo* and *in vitro* that the activated form of Src induces phosphorylation of c-Cbl and the consequent activation of its ligase activity that in turn promotes the ubiquitination and degradation of both c-Cbl itself and Src [9, 18]. In particular, on normal B cells Lyn inhibits BCR-mediated signaling events by phosphorylating and activating c-Cbl that in turn targets Lyn for ubiquitin dependent degradation [19, 20]. Based on this, we compared the ubiquitination and the phosphorylation status of Lyn and c-Cbl before and after BCR stimulation with anti-IgM and anti-IgD. We used a commercial kit permitting to pull-out and enrich all ubiquitinated proteins from lymphocyte lysate. The enriched protein population was then analyzed by WB and the ubiquitinated proteins were detected by primary antibody anti-Lyn and anti-c-Cbl. After detection of the proteins of interest, the blot was stripped and re-probed with anti-ubiquitin-HRP labeled antibody supplied in the kit (Ubi post stripping; Figure 2A). Data obtained from 10 independent experiments allowed to show that in CLL B cells, expressing both IgM and IgD (Supplementary Figure 2A), c-Cbl was not subjected to ubiquitination (Figure 2A, c-Cbl lane), despite its high level of phosphorylation on Y700 after stimuli (Figure 2B), one of the major phosphorylation sites of active form of c-Cbl [21, 22]. As regard Lyn which is constitutivly phosphorylated (Figure 2B, pLynY396 lane), we demonstrated that the protein was slightly ubiquitinated at steady state, but this state did not increase after anti-IgM and anti-IgD stimulation (Figure 2A). We obtained similar results using the approach of Proximity Ligation Assay (PLA) that allows *in situ* detection of protein interactions and modifications. Normal or CLL B cells were incubated with anti-Lyn or anti-Ubiquitin alone (as negative controls, not shown) or in combination with anti-Ubiquitin, processed following PLA protocol, and analyzed by confocal microscopy. Despite a basal level of Lyn ubiquitination revealed by the presence of green fluorescence spots, upon anti-IgM stimulation a specific induction of additional spots was observed only in normal B cells (Figure 2C). A successful BCR stimulation was assessed by intracellular Ca2+ influx after IgM and IgD (Supplementary Figure 2B i and ii). All together, these evidence suggest that upon BCR stimulation Lyn and c-Cbl are not subjected to negative regulation by ubiquitination in CLL B cells supporting a compromised protein homeostasis.

### c-Cbl does not associate with Lyn in neoplastic B cells

**Figure 3:**
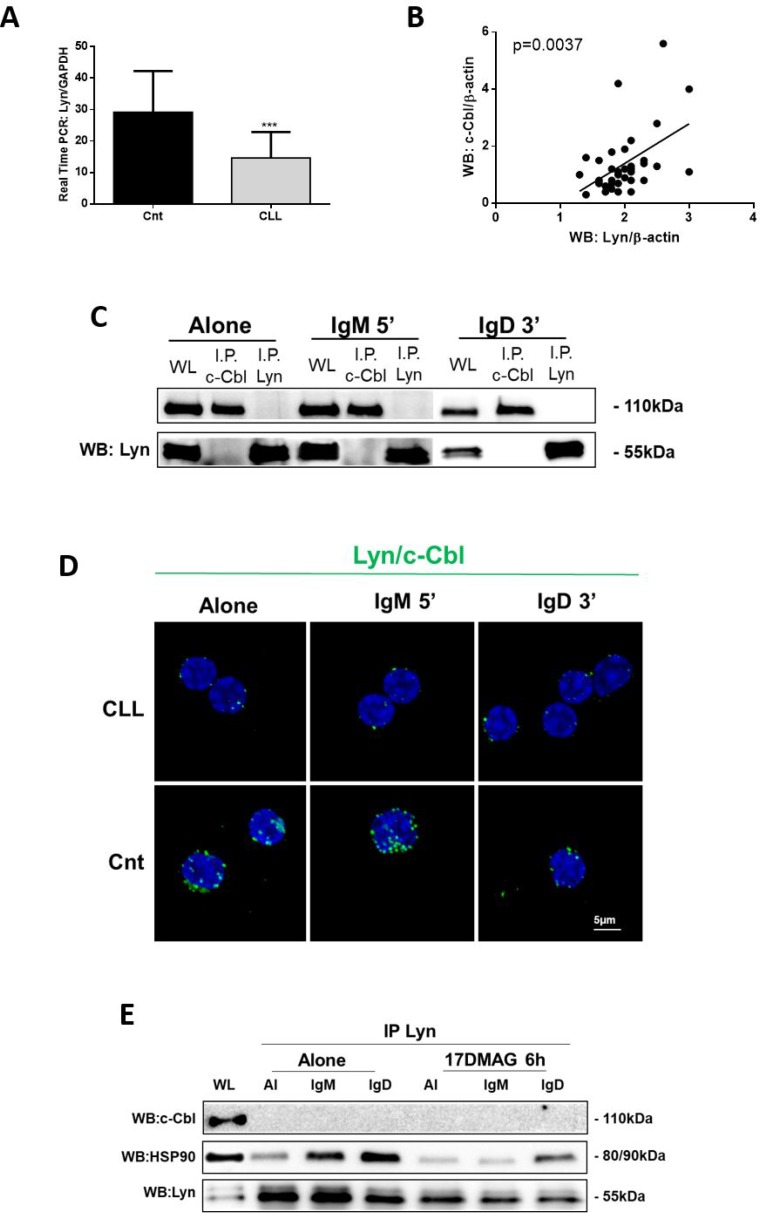
c-Cbl and Lyn were not associated neither after BCR engagement (**A**) Evaluation of mRNA Lyn expression levels by real-time PCR in 14 normal B lymphocytes (Cnt) and 23 neoplastic B cells (CLL). (**B**) Pearson correlation coefficient between Lyn and c-Cbl protein expression obtained from densitometric analysis of all 40 CLL B lymphocytes from CLL patients. (**C**) The lysates obtained from leukemic B cells of CLL patients (*n* = 15), before and after 5 minutes of IgM and 3 minutes of IgD stimuli (10 ng/mL), were immunoprecipitated with anti-Lyn (IP Lyn) and anti-c-Cbl antibody (IP c-Cbl). Immunocomplexes (IP) and whole lysate (WL) were loaded in SDS-PAGE (10% Acrylamide/Bis-acrylamide) and then probed with anti-c-Cbl and anti–Lyn. (**D**) PLA was performed on CLL and normal B cells (Cnt). Rabbit polyclonal anti-Lyn and mouse monoclonal anti-c-Cbl were used as primary Abs. Positive PLA signals are visualized as green fluorescent spots, nuclei are in blue (DAPI). Images are representative of three independent experiments, and were acquired with zoom 4 using 60X/1.35NA oil immersion objective. Z-projections of 15 slices are shown. Scale bar: 5 µm. (**E**) CLL B cells (*n* = 5) were treated for 6 h with 17-DMAG (100 µM) and the cytosol, isolated as in panel D of Figure 1, was immunoprecipitated with anti-Lyn antibody before and after anti-IgM (10 ng/mL) and anti-IgD (10 ng/mL) stimuli. Immunocomplexes were loaded in SDS-PAGE (10% Acrylamide/Bis-acrylamide) and then probed with anti-c-Cbl, anti-HSP90 and anti-Lyn.

We previously reported that Lyn kinase is 2.5- up to 5-fold over-expressed in leukemic with respect to normal B cells [23]. However, herein, we showed that Lyn mRNA is expressed in CLL lymphocytes (*n* = 23) at low levels respect to normal B cells (*n* = 14) (^***^p < 0.001; Figure 3A), suggesting the anomalous Lyn protein overexpression (^****^p < 0.0001; Supplementary Figure 3A) was not related to differences in gene transcription and/or mRNA stability but rather to a deregulation in Lyn turnover. We observed a positive and significant correlation between Lyn and c-Cbl protein levels at basal conditions (*n* = 40; *p* = 0.0037; Figure 3B). Hence, we performed co-immunoprecipitation experiments before and after BCR engagement upon IgM and IgD ligation, to evaluate whether protein over expression could favour Cbl/Lyn interaction. We already demonstrated that c-Cbl was not constitutive associate to Lyn, like in normal B cells [24]. Now, with our surprise, data obtained from 15 independent experiments allowed us to show that in CLL B cells, Lyn and c-Cbl were not associated even after BCR stimulation (Figure 3C), unlike from what happens in normal B lymphocytes [12, 14] (Supplementary Figure 1). We confirmed biochemical results using PLA assay (Cnt, Figure 3D).

We previously reported that Lyn is in an active conformation as integral component of an aberrant cytosolic 600-kDa multiprotein complex, associated with several proteins, such as Hsp90, HS1 and SHP-1L [24]. In particular, Hsp90 is tightly bound to cytosolic Lyn, thus stabilizing the aberrant complex and converting individual transient interactions into stable ones [24]. To rule out that this complex prevents the binding of c-Cbl to cytosolic Lyn, we treated neoplastic B-lymphocytes of 5 CLL patients with a potent HSP90 inhibitor, 17-DMAG, to dissociate the Lyn-Hsp90 complex. We used 17-DMAG for 6 h at the concentration of 500 nM, since under this condition we found a strong decrease in total Tyr phosphorylation level (Supplementary Figure 3B) without perturbing the cell viability (Supplementary Figure 3C). After 17-DMAG treatment we immunoprecipitated cytosolic Lyn and we found that neither before nor after IgM and IgD stimulation c-Cbl interacts with this kinase (Figure 3E). The lack of association between Cbl and Lyn support the conclusion that Cbl in not involved in Lyn protein turnover in CLL B cells.

### c-Cbl is constitutive associated with PI3Kp85 in CLL patients with basal phosphorylation at Y731

**Figure 4:**
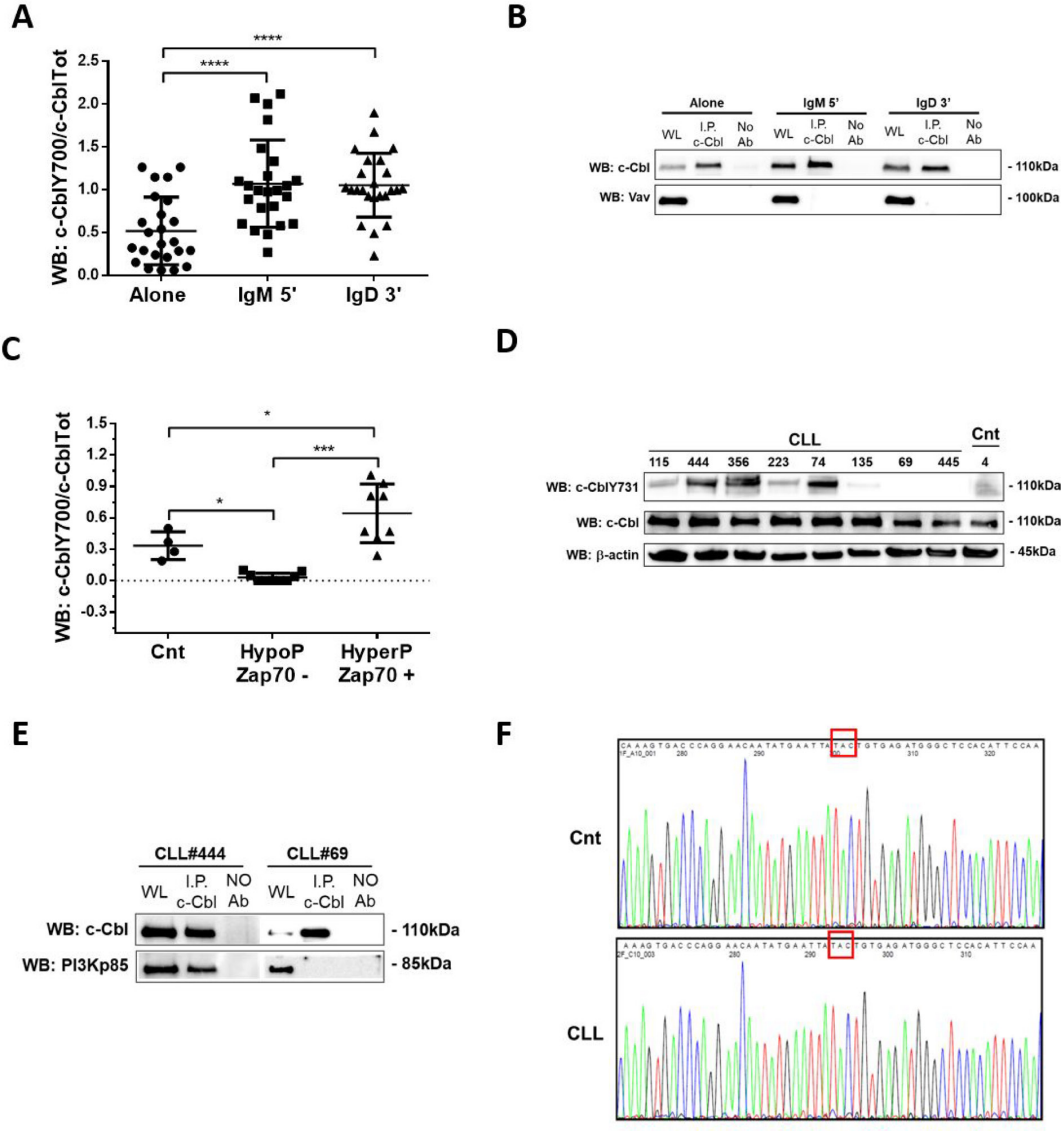
Analysis of c-Cbl phosphorylation and its association with PI3K regulatory subunit p85 (**A**) Densitometric ananlysis of c-CblY700/c-CblTot ratio in 24 CLL patients before (Alone) and after 5 minutes of IgM (IgM) and 3 minutes of IgD (IgD). Data obtained were evaluated for their statistical significance with pair Student’s *t* test (^****^p < 0.0001 between Alone and IgM and IgD). (**B**) The lysates obtained from 10 leukemic B cells before and after 5 minutes with IgM and 3 minutes with IgD stimuli (10 ng/mL) were immunoprecipitated with anti-c-Cbl. Immunocomplexes were loaded in SDS-PAGE (10% Acrylamide/Bis-acrylamide) and then probed with anti-c-Cbl and anti–Vav. (**C**) Densitometric analysis of c-CblY700/c-Cbl ratio in 4 healthy subjects (Cnt) and 20 CLL patients. The patients were divided in two groups, HypoP/Zap70– (*n* = 10) and HyperP/Zap70+ (*n* = 10) using as a cut-off the median of c-CblY700/c-Cbl ratio in normal controls. Data obtained were evaluated for their statistical significance with the Student’s *t* test (^*^p < 0.01 between Cnt and two groups of CLL patients; ^***^p < 0.001 between HypoP/Zap70– and HyperP/Zap70+ CLL groups). (**D**) Western blotting (10% Acrylamide/Bis-acrylamide) of 8 CLL (#115, #444, #356, #223, #74, #135, #69 and #445) and 1 normal B lymphocytes (Cnt) were representative of all 20 CLL and 5 Cnt samples analysed. The lysates obtained from normal B lymphocytes and leukemic B cells from CLL patients were analyzed by immunostaining with antibody against c-CblY731 and c-Cbl. Blots were reprobed with anti-β-actin antibody as loading control. (**E**) The lysates obtained from leukemic B cells of CLL patients (CLL#444 and CLL#69) were immunoprecipitated with anti-c-Cbl antibody. Immunocomplexes were loaded in SDS-PAGE (10% Acrylamide/Bis-acrylamide) and the membrane was probed with anti-c-Cbl and PI3Kp85 antibody. The figure is representative of 10 experiments. (**F**) Representative electropherograms of 20 CLL patients (CLL) and 5 normal controls (Cnt). Comparison of c-CBL LINKER-Ring region mutations (Exon 8) between normal controls (Cnt) and CLL patients (CLL). Red box highlights the hot spot mutation “TAC”.

Several studies have demonstrated the pivotal role of c-Cbl tyrosine phosphorylation for its adaptor function and E3 activity [11, 25, 26]. Among 22 tyrosine residues present in the molecule, we studied Y700 since it is one of the major phosphorylation sites of the “active” form of c-Cbl and docking site for Vav [21, 22]. Data obtained from 24 independent experiments showed that in CLL B cells, expressing IgM and IgD (Supplementary Figure 2A), the phosphorylation on Y700 increased after both IgM and IgD stimulus (Alone *vs* IgM and Alone *vs* IgD; ^****^p < 0.0001; Figure 4A and Supplementary Figure 4A), sustaining the involvement of c-Cbl in BCR signaling. However, despite a strong phosphorylation on Y700, we showed by co-immunoprecipitation assay that in neoplastic B lymphocytes, c-Cbl and Vav do not associate after stimulation with both IgM and IgD (Figure 4B), as described for normal B cells [21] (Supplementary Figure 1). These data supporting the hypothesis that in CLL other overexpressed proteins could bind the same phosphorylated site at higher affinity than Vav.

As already reported by Mankai and colleagues [10], we showed that at basal conditions the phosphorylation on Y700 is different among CLL patients. We divided our patients in two groups (Hypo-Phosphorylated and Hyper-Phosphorylated, Figure 4C) using as a cut-off the mean of Y700 levels detected in normal B cells (=0.21). Differently from what demonstrated by Mankai *et al.*, we highlighted that all the HypoP patients (*n* = 12) clustered in ZAP70–prognostic group; instead, all the HyperP patients (*n* = 12) were ZAP70+ (HypoP/Zap70− *vs* HyperP/Zap70+; ^***^p < 0.001, Student’s *t* test; Figure 4C). This evidence sustains the hypothesis that the basal phosphorylation on Y700 is maintained by overexpression and hyperactivation of different kinases (Zap70, Lyn and more others).

By WB we also analyzed the c-Cbl phosphorylation status at Y731, docking site for regulatory subunit p85 of PI3K (PI3Kp85), in 20 CLL patients and 5 normal controls. We demonstrated as some patients (*n* = 8; CLL444, CLL356, CLL74; Figure 4D) presented higher levels of Y731-phosphorylated at steady state respect to others with levels comparable to normal controls (*n* = 6; CLL115 and CLL223; Figure 4D) or with absent phosphorylation (*n* = 6; CLL135, CLL69 and CLL445; Figure 4D). This condition was independent from Zap70 and SHM prognostic groups (data not shown). Interestingly, unlike from what observed for Y700, we demonstrated a constitutive association between c-Cbl and PI3Kp85 in patients with higher phosphorylation at Y731 (CLL#444; Figure 4E). Instead, in those patients with lower or absent basal Y731-phosphorylation, c-Cbl and PI3Kp85 were not associated (CLL#69; Figure 4E). In some myelomonocytic leukemia patients the mutations of *cbl* gene at the region encoding for linker domain, enhanced the binding of c-Cbl to the PI3Kp85 [11] and the hyperphosphorylation at Y731. To assess whether *cbl* was mutated also in leukemic B cells, we sequenced the linker domain region in leukemic B cells of CLL patients. Among the 20 considered CLL cases, no mutations were found with respect to normal control (*N* = 3) (Figure 4F).

All these data suggest that in CLL c-Cbl although overexpressed only partially preserved the capability of binding to its canonical partners probably due to the presence of other overexpressed molecules and/or to its protein conformational status, since in some neoplastic B cells c-Cbl is hyperphosphorylated at basal conditions.

### c-Cbl is constitutively associated with Cortactin in those CLL patients with Cortactin overexpression

**Figure 5:**
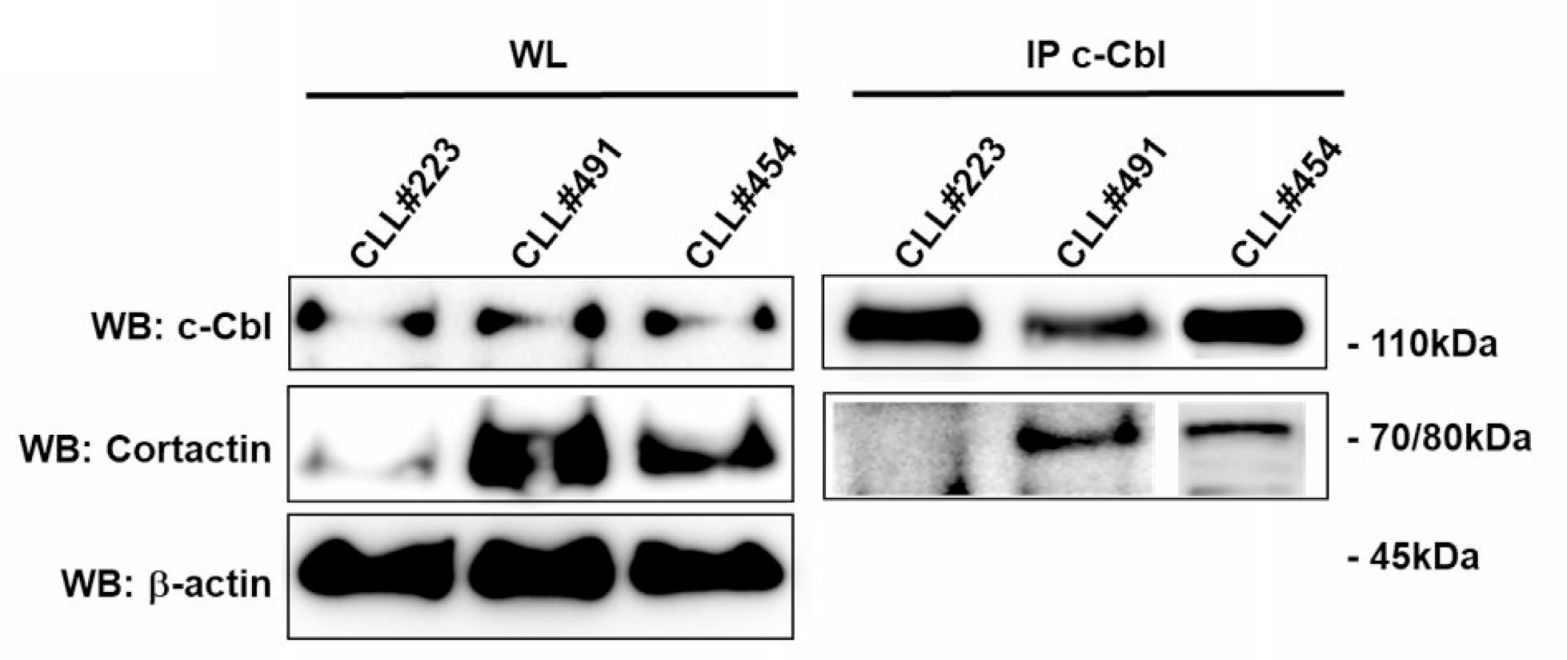
Analysis of c-Cbl and Cortactin association in CLL B lymphocytes The lysates obtained from leukemic B cells of CLL patients (*n* = 10) were immunoprecipitated with anti-c-Cbl (IP c-Cbl). Immunocomplexes (IP) and whole lysate (WL) were loaded in SDS-PAGE (10% Acrylamide/Bis-acrylamide) and then probed with anti-c-Cbl and anti-Cortactin antibody.

Several molecules inhibit Cbl proteins function without ubiquitination and degradation of the Cbl molecules. It is well known that after EGFR activation, Sprouty (Spry) proteins and Cdc42 binding to Cbl sequester it away from the EGFR, thus inhibiting its activity [27]. Other proteins (such as Cortactin, HPV16 E5, Alix and SHIP2) prevent Cbl-mediated downregulation of the EGFR without degradation of the Cbl protein, although the mechanisms by which they inhibit Cbl function are not clear [28, 29]. Recently, in colorectal cancer (CRC) Cortactin overexpression inhibited the ubiquitin-mediated degradation of EFGR by suppressing the coupling of c-Cbl with EGFR [30]. Since we previously demonstrated that Cortactin is a Lyn substrate and its overexpression is related to bad prognosis and more invasiveness/metastasis in CLL [5, 31], herein we investigated its relationship with c-Cbl. As it is appreciable in Figure 5, in CLL patients with Cortactin overexpression, c-Cbl and Cortactin were constitutive associated (CLL#491 and CLL454; Figure 5) while, in patients with lower Cortactin expression the two proteins did not co-immunoprecipitate (CLL#223; Figure 5), independently from c-Cbl protein level. Therefore, Cortactin could probably influence c-Cbl activity by supporting its adaptor function and by promoting tumorigenesis.

## DISCUSSION

This study provides novel insights into c-Cbl role in CLL cells. We demonstrated that in CLL B cells c-Cbl has a role as an adaptor protein rather than E3 ubiquitin ligase activity, supporting pro-survival signaling from BCR. c-Cbl is inactive in unstimulated cells and upon interaction with an active kinase its E3 activity is induced, leading to ubiquitination and downregulation of the target kinase and c-Cbl itself [32, 33]. c-Cbl induces ubiquitination of a number of non-receptor protein tyrosine kinases (e.g. Syk, Fyn, Lck, Fgr and Lyn) [22, 34]. It has been demonstrated that c-Cbl differentially modulates the BCR-mediated signaling pathways through the targeting of Lyn by ubiquitination, thus affecting B cell development [16]. In fact, Lyn kinase expression and activity is up-regulated in c-Cbl^−/−^ B cells, which correlates inversely with Lyn ubiquitination. On the contrary, we highlighted that in CLL B cells both c-Cbl and Lyn were overexpressed and there was a positive correlation between expression levels of the two proteins. However, we demonstrated that Lyn and c-Cbl did not associate upon BCR engagement, neither when cells were treated with 17-DMAG releasing Lyn from aberrant 600-kDa cytosolic multiprotein complex [24]. In the modulation of signals initiated by Src tyrosine kinases, it is necessary that c-Cbl forms a complex with the target protein, promoting the binding of ubiquitin molecules and degradation of the complex via proteasome [20, 35]. The lack of interaction between Lyn and c-Cbl suggests the latter is not involved in Lyn turnover in neoplastic B-lymphocytes. Further investigations will be necessary in order to clarify how Lyn is overexpressed in CLL B cells even though mRNA levels are lower than those of normal B cells. Moreover, we also demonstrated that in CLL B lymphocytes c-Cbl was not ubiquitinated and this might be the reason why its overexpression is preserved.

In normal B cells, CIN85 is poorly expressed, constitutively associated to c-Cbl and is required for Cbl-mediated regulation of BCR signaling and for downstream events such as survival, growth, and differentiation of human B cells [36]. CIN85 acts as a key scaffolding adaptor that permits the spatial proximity of Src-family protein kinases and Cbl proteins facilitating c-Cbl activity [14]. However, in neoplastic B cells we demonstrated a lack of interaction between CIN85 and c-Cbl before and after BCR stimulation. This lack of association could be likely due to the presence of specific CIN85 mRNA spliced forms in CLL B cells. Indeed, we noticed that, in normal B cells, CIN85 migrated in SDS-PAGE as a band of 85kDa, while in CLL B lysates it migrates as two distinct bands of about 80 and 85kDa. To this regard, it has been demonstrated that various molecular forms of CIN85, encoded by specifically spliced mRNA, correspond to the full-length form or different combination of SH3 domains as well as additional forms that are the products of post-translational modifications [37, 38]. Moreover, using MCF-7 human breast adenocarcinoma cells and cell fractionation technique, it has been demonstrated a specific association of CIN85 molecular forms with different subcellular compartments. Induction of apoptosis in these cells triggers changes in both content and subcellular distribution of CIN85 molecular forms and these changes are unique for each form under different types of apoptosis [37]. Regardless of the responsible mechanism(s), we speculated that the lack of association between Cbl and CIN85 in CLL B cells could influence the binding of c-Cbl to Lyn kinase and its turnover.

Protein homeostasis is critical for the processes that govern cell survival, thus the aberrant signaling by protein tyrosine kinases has been associated with many human cancers. The escape from c-Cbl mediated down-regulation has been shown to be an important event in receptor deregulation [39]. Activation of the E3 activity of c-Cbl is driven by tyrosine phosphorylation and Y700 is one of the 22 tyrosine residues of the molecules and one of the major phosphorylation sites of active form of c-Cbl. Mankai *et al.* showed that Y700 was lower phosphorylated in advanced BINET stage B CLL patients and that the disease progression was correlated with a specific threshold value of optical density ratio of ZAP70/c-Cbl.P: stage A patients were found to have a ratio <1 while stage B patients were shown to have a ratio ≥1. They supposed that disease progression might result from loss of negative regulation, so that intracellular milieu becomes permissive to deregulate positive signals selected by CLL B cells for survival purpose. On the contrary, we did not find any correlation between Y700 expression levels and BINET stage (data not show) and we demonstrated that basal phosphorylation at Y700 were higher in patients ZAP70+ with a bad prognosis. This evidence sustains the hypothesis that the c-CblY700 basal phosphorylation is maintained by overexpression and hyperactivation of different kinases, and that it may contribute to stabilize c-Cbl in its “active” form. The lack of interaction between c-Cbl and Vav after BCR engagement, although a strong Y700 phosphorylation was detected, contributes to sustain the hypothesis that c-Cbl conformation in CLL B cells was altered thus influencing its activity.

Interaction with PTKs leads to phosphorylation of a highly conserved tyrosine in the linker helix region of c-Cbl protein and this event is required for its ligase activity [40–42]. Src kinases have been shown to phosphorylate Cbl on Y371 and trigger interdependent ubiquitination and degradation of both proteins [9]. This phosphorylation releases c-Cbl from its autoinhibited structure by triggering a conformational change that leads to an enhanced tyrosine phosphorylation firstly on Y700 and then Y731 site, and an efficient transfer of ubiquitin from the E2 enzyme to the substrate proteins. Sporadic and germline c-CBL mutations have been yet identified in JMML (Juvenile Myelomonocytic Leukemia) patients with the emerging of Y371H mutation, which results in the loss of Cbl’s ubiquitin ligase function. In particular, c-CblY371H, with respect to WT c-Cbl, promotes stabilization of the otherwise labile phosphorylated Src, which in turn increases the phosphorylation at Y700, Y744 and Y731 residues of stable mutant c-Cbl(Y371H). Moreover, cells expressing c-CblY371H are chemoresistant due to hyperactive Src kinase that promotes AKT signaling via enhanced binding of c-Cbl(Y371H) to the PI3K regulatory subunit p85 [26, 43]. We demonstrated that c-Cbl was hyperphoshorylated at Y731 in those patients with constitutive association to PI3Kp85 and this condition is probably due to structural protein modification since we did not find any mutations on linker-RING region in CLL patients. These results support the idea that c-Cbl in CLL neoplastic cells has a particular protein structure that influences its interaction with other proteins and thus its function. We proposed a model for CLL in which overexpressed c-Cbl associates to regulatory subunit p85 of PI3K maintaining active the catalytic subunit p110 of PI3K, and in turn, the signal propagated from BCR.

Recently, in colorectal cancer (CRC) Cortactin overexpression has been correlated with inhibition of ubiquitin-mediated degradation of EFGR by suppressing the coupling of c-Cbl with EGFR [30]. We previously demonstrated that Cortactin was overexpressed in CLL patients with bad prognosis [31] and that this overexpression was associated to more invasiveness and response to chemotactic stimuli [5]. Herein, we demonstrated that c-Cbl was constitutive associated to Cortactin in those CLL patients with Cortactin overexpression, suggesting that this interaction could interfere with c-Cbl activity. Further investigations are required to better understand how c-Cbl and Cortactin could play together in the altered BCR or CXCR4/CXCL12 axis signaling.

All together, these data support our hypothesis that, in CLL, c-Cbl has adaptor function rather than E3 ligase activity. In CLL cells, the absence of the association of c-Cbl to CIN85 and Lyn, also after BCR triggering, together with the constitutive phosphorylation at Y731 and association to Cortactin in those patients in which Cortactin is highly expressed, sustain the hypothesis that c-Cbl has an altered protein structure that influences its activity and in turn cell homeostasis.

## MATERIALS AND METHODS

### Patients, cell purification and reagents

We analysed peripheral blood cells of 13 healthy donors and 40 patients diagnosed with CLL treatment-naïve. The purity of the obtained peripheral B cells was at least 95% (CD19+), as assessed by flow cytometry (FC). For the study we considered pathologic sample with at least 95% of purified CD19+/CD5+. Informed consent was obtained in accordance to Declaration of Helsinki. Patient characteristics are reported in Supplementary Table 1. Cells were separated from peripheral blood as detailed in the Supplementary Data. Polyclonal goat F(ab’)2 fragments to human IgM or to human IgD L chain (Southern Biotechnology, Birmingham, AL, USA) were used for cell stimulation (anti-IgM and anti-IgD). Anti-c-Cbl antibody, anti-c-Cbl (phospho Y700 and phospho Y731) and anti-PMCA antibody were purchased from Abcam (Cambridge, UK), anti-CIN85 and anti-pTyr from Millipore (Burlington, Massachusetts, USA), anti-Vav, anti-PARP and anti-PI3Kp85 from CellSignaling (Danvers, Massachusetts, USA), anti-Lyn from SantaCruz (Dallas, Texas, USA), anti-ubiquitin from Enzo Lifescience (Saint Louis, USA), finally, anti-β-Actin from Sigma (Saint Louis, USA).

### Flow cytometry analysis (FC)

Purified populations (CD5+/CD19+ leukemic cells and CD19+ normal B lymphocytes) were analysed for purity and viability by FC analysis. Surface staining was performed with FITC-CD5, APC-CD19 (BD Bioscience, Franklin Lakes, New Jersey, USA), FITC- and PE-conjugated goat polyclonal H chain–specific anti-IgM and anti-IgD Abs respectively (DAKO, Agilent, Santa Clara, California, USA). Purified samples were acquired by FACS Canto II cytometer (Becton Dickinson) and data were processed using DIVA and FlowJo Softwares (Becton Dickinson).

### Intracellular calcium measurement by Flow Cytometry

For measurement of intracellular calcium mobilisation, a total of 1 × 10^7^ cells were incubated in 1 ml complete RPMI with 4 μM Fluo-4-AM (Invitrogen, Carlsbad, Califonia, USA) at 37° C for 30 min. Cells were then resuspended at 5 × 10^6^/ml and 100 μl of cell suspension was added into 400 μl of prewarmed RPMI. Cells were analysed by FACS CANTO II flow cytometer (Becton Dickinson). After 30 s of baseline acquisition, α-IgM F(ab’)2 and α-IgD F(ab’)2 (10 μg/ml) (Southern Biotech) were added and the fluorescence intensity (FI) was recorded for 5 min. Then, was added Ionomycin (1µg/mL) and FI was recorded for other 2 minutes. For quantification, mean baseline FI was subtracted from the peak intensity after stimulation and divided for the value obtained subtracting mean baseline FI to mean Ionomycin FI. The resulting value was termed “calcium response”.

### Cell viability testing

Apoptosis was assessed using the Annexin V Apoptosis Detection Kit accordingly to the manufacturer’s instructions (Becton Dickinson). Briefly, after incubation with 17-DMAG (Selleckem, Huston, TX, USA) (100 nM or 500 nM) for 4 h or 6 h, aliquots of 250.000 cells were harvested, washed and incubated for 10 min in the dark at room temperature with 100 µl of binding buffer, 5 µl of Annexin V-fluorescein isothicyanate (FITC), and 3 µl of propidium iodide (PI). Cells were analysed by FACS CANTO II flow cytometer (Becton Dickinson).

### Subcellular protein fractionation

Nuclear/Cytoplasmic/Membrane fractionations have been performed with the Subcellular Protein Fractionation Kit for Cultured Cells accordingly to the manufacturer’s instructions (Thermo Fisher Scientific; Waltham, MA, USA).

### Lyn mRNA expression by real-time PCR analysis

The primers used to evaluate Lyn and β-Actin mRNA levels are: Lyn F 5′-GCT CAG ATT GCA GAG GGA ATG-3′ and R 5′-GAG CCG TCC ACT TAA TAG GGA-3′; β-actin F 5′-CCA GCT CAC CAT GGA TGA TG-3′ and R 5′-ATG CCG GAG CCG TTG TC-3′. Procedure is detailed in Supplementary data.

### Western blotting analysis (WB)

Cells from CLL patients and healthy donors were prepared by cell lyses as previously described [4] and it is detailed in Supplementary data.

### Ubiquitination detection kit

Signal-Seeker^™^ kits use affinity beads to pull-out and enrich modified proteins from cell lysate according to the manufacturer’s instructions (Cytoskeleton, Inc, Denver, CO, USA). The enriched protein population was then analyzed by standard WB procedures and the modified protein of interest is detected by the end-user using their own primary antibody.

### Proximity ligation assay (PLA) and confocal microscopy

B cells were plated on poly-L-lysine-coated multichamber slides (LabTek, Thermo Scientific) and let adhere for 30 minutes at 37° C. Cells were then fixed with 4% paraformaldehyde and permeabilized with 0.1 Triton-X-100 for 5 min. Proximity Ligation Assay was performed using Duolink PLA *In Situ* Green Starter Kit (SigmaAldrich, Mouse/Rabbit) according to manufacture’s instructions, as previously described [44]. Anti-Lyn, anti-Cbl and anti-ubiquitin were used as primary Abs. High-resolution images (800 × 800 pixel, 8 μs/pixel) were acquired at room temperature using IX83 FV1200 MPE laser-scanning confocal microscope with a 60×/1.35 NA UPlanSAPO oil immersion objective (all from Olympus), and images were processed with Fiji ImageJ software.

### Statistical analysis

Statistical analysis was performed using Student’s *t* test, paired Student’s *t* test and ANOVA test. Data were expressed as means ± Standard Deviation (SD) and were considered statistically significant when *p* values were ^*^< 0.05, ^**^< 0.01, ^***^< 0.001 and ^****^< 0.0001.

## SUPPLEMENTARY MATERIALS FIGURES AND TABLE



## Abbreviations

BCR: B-Cell Receptor
c-Cbl: c-Casitas B lineage lymphoma
CLL: Chronic Lymphocytic Leukemia
