# Supplementary material for: Abnormal regulation of BCR signalling by c-Cbl in chronic lymphocytic leukaemia
Source: Oncotarget. 2018 Aug 14;9(63):32219–31. doi: 10.18632/oncotarget.25951 (PMC6114956; doi:10.18632/oncotarget.25951)
Supplement: Supplementary file 1 [file oncotarget-09-32219-s001.pdf]

Abnormal regulation of BCR signalling by c-Cbl in chronic lymphocytic leukaemia

SUPPLEMENTARY MATERIALS

Patients, cell purification and reagents

We analysed peripheral blood cells from 13 healthy donors and 40 patients with treatment-naïve CLL. The purity of the obtained peripheral B cells was at least 95% (CD19+), as assessed by flow cytometry (FC). For the study we considered pathologic sample with at least 95% of purified CD19+/CD5+. Informed consent was obtained in accordance to Declaration of Helsinki. Patient characteristics are reported in Supplementary Table 1. Cells were separated from peripheral blood as detailed in the Supplementary Data. Polyclonal goat F(ab')2 fragments to human IgM or to human IgD L chain (Southern Biotechnology, Birmingham, AL, USA) were used for cell stimulation (anti-IgM and anti-IgD). Anti-c-Cbl antibody, anti-c-Cbl (phospho Y700 and phospho Y731) and anti-PMCA antibody were purchased from Abcam (Cambridge, UK), anti-CIN85 and anti-pTyr from Millipore (Burlington, Massachusetts, USA), anti-Vav, anti-PARP and anti-PI3Kp85 from CellSignaling (Danvers, Massachusetts, USA), anti-Lyn from SantaCruz (Dallas, Texas, USA), anti-ubiquitin from Enzo Lifescience (Saint Louis, USA), finally, anti-β-Actin from Sigma (Saint Louis, USA).

Cell separation

We obtained peripheral blood from 40 untreated patients that satisfied standard morphological and immunophenotypic criteria for CLL. Peripheral blood mononuclear cells (PBMCs) of the patients were isolated by density-gradient centrifugation over Ficoll-Hypaque (Amersham Biosciences; Buckinghamshire, UK). Where necessary, further purifications were performed using the RosetteSep isolation kit for B-cells (STEMCELL Technologies; Vancouver, Canada). Finally, all the samples utilized had a CD19+ B-cell content greater than 95%. Untreated peripheral blood B-cells were isolated from PBMCs of 13 normal donors, representative of the adult healthy population, using the RosetteSep isolation kit for B-cells. The purity of the obtained peripheral blood B-cells was at least 95% (CD19+), as assessed by FC.

Flow cytometry analysis (FC)

Purified populations (CD5+/CD19+ leukemic cells and CD19+ normal B lymphocytes) were analysed for purity and viability by FC analysis. Surface staining was performed with FITC-CD5, APC-CD19 (BD Bioscience, Franklin Lakes, New Jersey, USA), FITC- and PE-conjugated goat polyclonal H chain–specific anti-IgM and anti-IgD Abs respectively (DAKO, Agilent, Santa Clara, California, USA). Purified samples were acquired by FACS Canto II cytometer (Becton Dickinson) and data were processed using DIVA and FlowJo Softwares (Becton Dickinson).

Intracellular calcium measurement by Flow Cytometry

For measurement of intracellular calcium mobilisation, a total of 1×10^7 cells were incubated in 1 ml complete RPMI with 4 µM Fluo-4-AM (Invitrogen, Carlsbad, Califonia, USA) at 37° C for 30 min. Cells were then resuspended at 5×10^6/ml and 100 µl of cell suspension was added into 400 µl of prewarmed RPMI. Cells were analysed by FACS CANTO II flow cytometer (Becton Dickinson). After 30 s of baseline acquisition, α-IgM F(ab')2 and α-IgD F(ab') 2 (10 µg/ml) (Southern Biotech) were added and the fluorescence intensity (FI) was recorded for 5 min. Then, was added Ionomycin (1 µg/mL) and FI was recorded for other 2 minutes. For quantification, mean baseline FI was subtracted from the peak intensity after stimulation and divided for the value obtained subtracting mean baseline FI to mean Ionomycin FI. The resulting value was termed "calcium response".

Cell viability testing

Apoptosis was assessed using the Annexin V Apoptosis Detection Kit accordingly to the manufacturer's instructions (Becton Dickinson). Briefly, after incubation with 17-DMAG (Selleckem, Huston, TX, USA) (100nM or 500nM) for 4h or 6h, aliquots of 250.000 cells were harvested, washed and incubated for 10 min in the dark at room temperature with 100 µl of binding buffer, 5 µl of Annexin V-fluorescein isothicyanate (FITC), and 3 µl of propidium iodide (PI). Cells were analysed by FACS CANTO II flow cytometer (Becton Dickinson).

Subcellular protein fractionation

Nuclear/Cytoplasmic/Membrane fractionations have been performed with the Subcellular Protein Fractionation

Kit for Cultured Cells accordingly to the manufacturer's instructions (Thermo Fisher Scientific; Waltham, MA, USA). The Subcellular Protein Fractionation Kit for Cultured Cells contains four extraction buffers, a stabilized nuclease and Thermo Scientific Halt Protease Inhibitor Cocktail. Each kit has enough reagents to fractionate 50 cell pellets of 20 µL each, equivalent to approximately 2 g of cell paste. The first kit reagent, when added to a cell pellet, causes selective membrane permeabilization, releasing soluble cytoplasmic contents. The second reagent dissolves plasma, mitochondria and ER-golgi membranes but does not solubilize the nuclear membranes. After recovering intact nuclei by centrifugation, a third reagent yields the soluble nuclear extract.

Lyn mRNA expression by real-time PCR analysis

Total cellular RNA from patient samples were extracted from 5–10 × 10^6 leukemic cells using RNeasy Mini Kit (Qiagen; Hilden, Germany), according to the manufacturer's protocol and treated with DNase (Qiagen). First strand complementary DNA (cDNA) was generated from 1 µg total RNA using oligo-dT primer and the AMV reverse transcriptase (Reverse Transcription System, Promega Corporation; Madison, WI). Real-Time quantitative PCR amplifications reaction were carried out in an ABI Prism 7000 sequence detection system (Applied Biosystems; Foster City, CA) in a 15 µl volume. SYBR Green 3 PCR Master Mix was purchased from Applied Biosystems (P/N 4309155), containing ampliTaq Gold DNA Polymerase and optimized buffer components. A fraction of 5 µM primers and 1,5 µl of cDNA were added to SYBR Green master mix to make a final 15 µl reaction volume. The primers used for Lyn and β-Actin amplifications are: Lyn Forward Lyn F 5′- GCT CAG ATT GCA GAG GGA ATG -3′ and R 5′- GAG CCG TCC ACT TAA TAG GGA -3′; β-actin F 5′- CCA GCT CAC CAT GGA TGA TG -3′ and R 5′- ATG CCG GAG CCG TTG TC -3′. These primers were obtained using the Primer Express computer software (Applied Biosystems). PCR reactions were performed under the following conditions: initial denaturation at 95° C for 10 min followed by 95° C for 15s and 60° C for 1s cycled 45 times. Each quantization target was amplified in duplicate samples. A no template control for each master mix and two standard curves were generated for Lyn and β-actin using Jurkat cDNA in a serial dilution 1, 1:5, 1:25 and 1:125. The relative amounts of mRNA was determined by comparison with standard curves. For each sample, results were normalized for β-actin expression. To distinguish specific amplicons from non-specific amplifications, a dissociation curve was generated.

Immunoprecipitation

Cell extract (20 × 10^5 for each assay) from CLL patients and normal controls were incubated with the antibody for the protein of interest (anti-c-Cbl, anti-CIN85 and anti-Lyn, see "Patients, cell purification and reagents" section) to permit the binding to the protein in solution. The antibody/antigen complex is then pulled out of the sample using protein A/G-coupled agarose beads (Santa Cruz Biotech. CA). This isolates the protein of interest from the rest of the sample. The sample can then be separated by SDS-PAGE for western blot analysis.

Western blotting analysis (WB)

Cells (5 × 10^5 for each assay) from CLL patients and normal controls were prepared by cell lyses with Tris 20 mM, NaCl 150 mM, EDTA 2 mM, EGTA 2 mM, Triton X-100 0.5% supplemented with complete protease inhibitor cocktail (Roche; Mannheim, Germany) and sodium orthovanadate 1 mM (Calbiochem; Gibbstown, NJ). Samples were then subjected to SDS/PAGE (7,5% or 10% gels), transferred to nitrocellulose membranes, immunostained with Anti-c-Cbl antibody, anti-c-Cbl (phospho Y700 and phospho Y731) and anti-PMCA antibody were purchased from Abcam (Cambridge, UK), anti-CIN85 and anti-pTyr from Millipore (Burlington, Massachusetts, USA), anti-Vav, anti-PARP and anti-PI3Kp85 from CellSignaling (Danvers, Massachusetts, USA), anti-Lyn from SantaCruz (Dallas, Texas, USA), anti-ubiquitin from Enzo Lifescience (Saint Louis, USA), finally, anti-β-Actin from Sigma (Saint Louis, USA). Blots were revealed using an enhanced chemiluminescent detection system (Amersham Biosciences; Little Chalfont, UK) and were acquired with the CHEMI DOC XRS supply (Bio-Rad; Hercules; CA; USA) and analysed by Image J launcher software.

Ubiquitination detection kit

Signal-Seeker™ kits use affinity beads to pull-out and enrich modified proteins from cell lysate according to the manufacturer's instructions (Cytoskeleton, Inc, Denver, CO, USA). The enriched protein population was then analysed by standard WB procedures and the modified protein of interest is detected by the end-user using their own primary antibody.

The Ubiquitination Affinity Beads (Ubiquitination Affinity Beads 1: Cat #UBA01) contain crosslinked Ubiquitin Binding Domains (UBDs), also called Ubiquitin-associated Domains (UBAs) and, in cases where multiple domains are expressed in a single protein, Tandem Ubiquitin Binding Entities (TUBES). UBDs have the unique characteristic of capturing both monoubiquitinated and polyubiquitinated proteins with high affinity. As mono-, multi– and poly-ubiquitination often confer unique, non-redundant properties to their target protein it is crucial to obtain the complete ubiquitination profile of any given target protein.

Proximity ligation assay (PLA) and confocal microscopy

B cells were plated on poly-L-lysine-coated multichamber slides (LabTek, Thermo Scientific) and let adhere for 30 minutes at 37° C. Cells were then fixed with 4% paraformaldehyde and permeabilized with 0.1 Triton-X-100 for 5 min. Proximity Ligation Assay was performed using Duolink PLA *In Situ* Green Starter Kit (SigmaAldrich, Mouse/Rabbit) according to manufacture's instructions, as previously described[13]. Anti-Lyn, anti-Cbl and anti-ubiquitin were used as primary Abs. High-resolution images (800 × 800 pixel, 8 µs/pixel) were acquired at room temperature using

IX83 FV1200 MPE laser-scanning confocal microscope with a 60×/1.35 NA UPlanSAPO oil immersion objective (all from Olympus), and images were processed with Fiji ImageJ software.

Sanger sequencing

For identification of c-CBL mutations we used the upstream primer 5′-TGGCTTATGTGAACCAACTCC–3′ (CBLexon7F), and downstream primer 5′–CTTGAGGGAACACATACTCG–3′ (CBLexon10R). Total RNA was isolated by means of RNasi Mini Kit (QIAGEN, Milano) from 9×10^6 B lymphocytes fractions prepared from anticoagulated peripheral blood. Reverse transcription to complementary DNA (cDNA) was carried out with Promega kit in a final volume of 20 μL according to the manufacturer's protocol, and cDNA was ready for automated DNA sequencing analysis. The reaction conditions were as follows: 95° C for 1 minute, 60° C for 1 minute, and 72° C for 1 minutes for 35 cycles. DNA was sequenced using dye terminator technology and an ABI 3130 sequencer (Applied Biosystems).

Statistical analysis

Statistical analysis was performed using Student's *t* test, paired Student's *t* test and ANOVA test. Data were expressed as means ± Standard Deviation (SD) and were considered statistically significant when *p* values were $^{*}<0.05$, $^{**}<0.01$, $^{***}<0.001$ and $^{****}<0.0001$.

Supplementary Table 1: Patients characteristics

Patients (number)	40
Age, mean (range)	65 (40–89)
Male/Female	27/20
WBC × 10^9/L, mean (range)	49789 (3000–50000)
% Lymphocytes, mean (range)	74 (24–97)
SHM mutated[1]/unmutated	14/26
CD38[2] positive/negative	12/22
ZAP70[3] positive/negative	17/17

1) SHM: Somatic HyperMutation; cut off < 98%;
2) Cut off ≥ 30%;
3) ZAP70: Zeta-Associated Protein 70; cut off ≥ 0.5;
WBC: White Blood Cell.

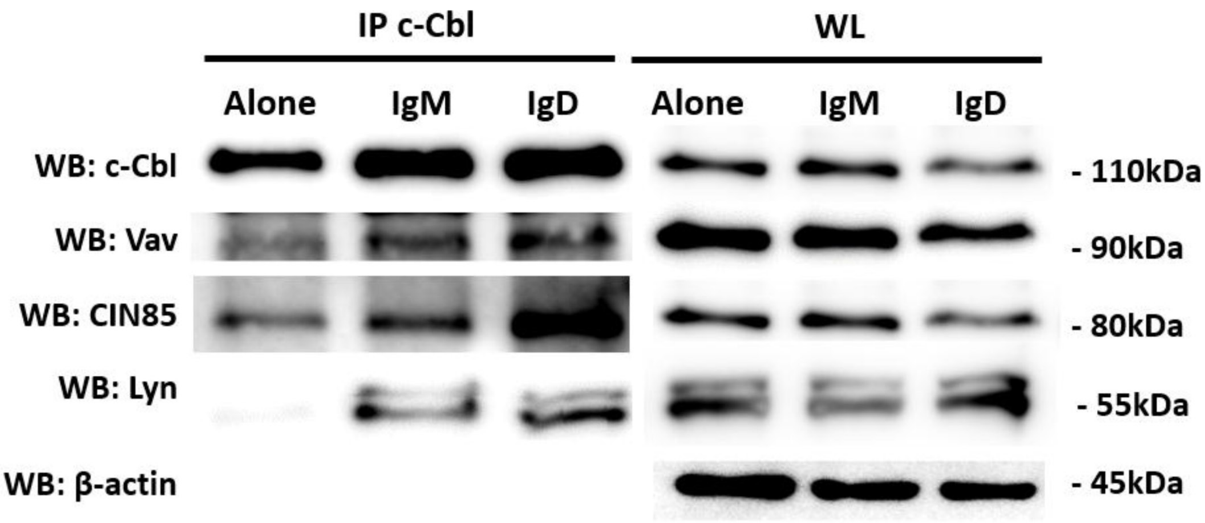

Supplementary Figure 1: c-Cbl associates with CIN85, VAV and Lyn after BCR engagement in normal B lymphocytes. Protein lysates of normal B cells enriched from 3 buffy coat, before and after 5 minutes of IgM and 3 minutes of IgD stimuli (10 ng/mL), were immunoprecipitated with anti-c-Cbl (IP c-Cbl). Immunocomplexes (IP) and whole lysate (WL) were loaded in SDS-PAGE (10% Acrylamide/ Bis-acrylamide) and then probed with anti-c-Cbl, anti–Lyn, anti-CIN85, anti-VAV. Anti-β-actin was used only for WL.

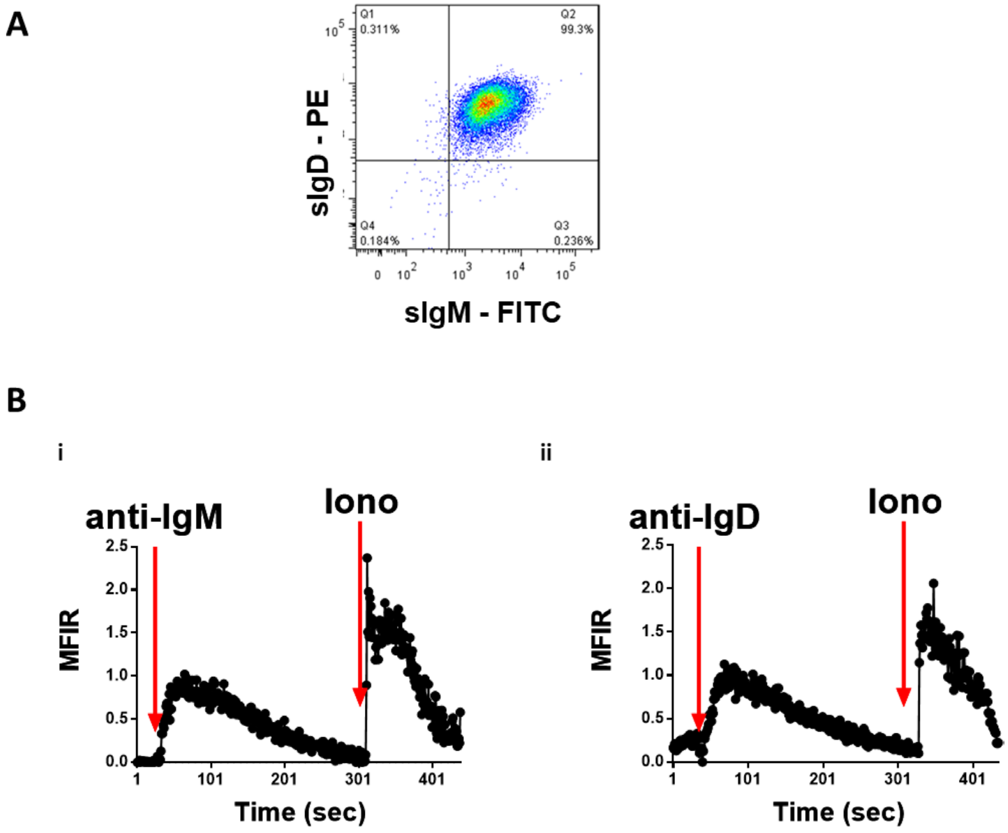

Supplementary Figure 2: IgM and IgD expression and calcium mobilization in CLL B analyzed by flow cytometry. (**A**) Representative plot for IgM and IgD expression (MFI) of 20 CLL patients. (**B**) The relative mean fluorescence intensity (MFIR) of intracellular calcium is plotted as a function of time (sec): arrows indicates IgM (i), IgD (ii) and ionomycin (Iono) stimulation.

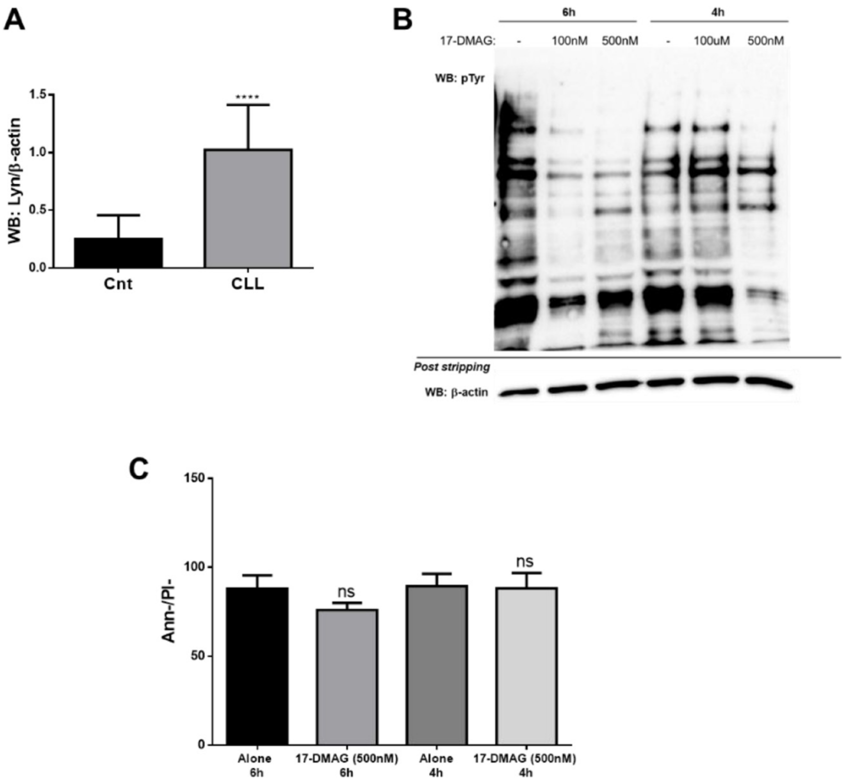

Supplementary Figure 3: Lyn expression in CLL B lymphocytes and 17-DMAG treatment. (**A**) Figure represents the densitometric value (arbitrary unit) of Lyn/β-actin ratio of B cells samples of 40 CLL patients (CLL) and 13 healthy subjects (Cnt). (**B**) Representative Western Blotting (10% Acrylamide/Bis-acrylamide) of CLL B lymphocytes after 17-DMAG treatment. The lysates obtained from leukemic B cells from 5 CLL patients were treated for 6 h and 4 h after 17-DMAG at the concentration of 100 nM and 500 nM. After SDS-PAGE, we analyzed the membrane by immunostaining with antibody against pTyr. Blots were reprobed with anti-β-actin antibody as loading control post stripping. (**C**) Viability of leukemic B cells cultured alone and in presence of 17-DMAG (100 nM or 500 nM) was assessed after 4 h and 6 h by FC using AnnexinV/PI staining. Data are presented as mean ± SD for 5 separated experiments; p = ns; paired Student's *t* test.

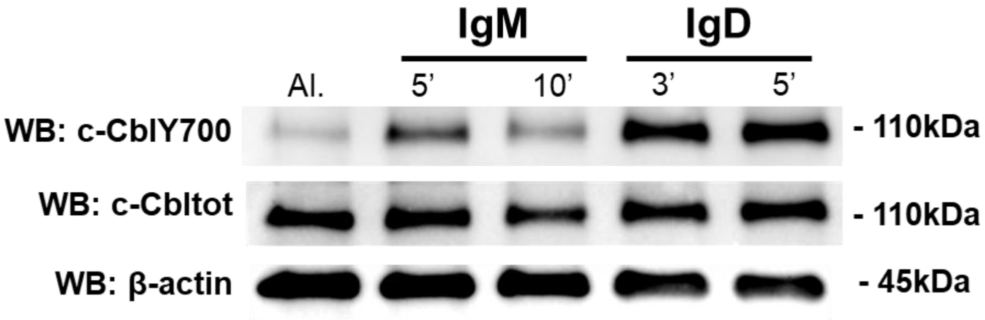

Supplementary Figure 4: Evaluation of c-Cbl on Y700 phosphorylation site. A) Western blotting (10% Acrylamide/Bis-acrylamide) of CLL B lymphocytes is representative of all 24 samples analysed. The lysates obtained from leukemic B cells, before and after IgM (5′ and 10') and IgD (3′ and 5′), were analyzed by immunostaining with antibody against pCblY700, c-Cbltot and β-actin.
